# Classifying Chinese Questions Related to Health Care Posted by Consumers Via the Internet

**DOI:** 10.2196/jmir.7156

**Published:** 2017-06-20

**Authors:** Haihong Guo, Xu Na, Li Hou, Jiao Li

**Affiliations:** ^1^ Institute of Medical Information & Library, Chinese Academy of Medical Sciences Beijing China

**Keywords:** classification, natural language processing, hypertension, consumer health information

## Abstract

**Background:**

In question answering (QA) system development, question classification is crucial for identifying information needs and improving the accuracy of returned answers. Although the questions are domain-specific, they are asked by non-professionals, making the question classification task more challenging.

**Objective:**

This study aimed to classify health care–related questions posted by the general public (Chinese speakers) on the Internet.

**Methods:**

A topic-based classification schema for health-related questions was built by manually annotating randomly selected questions. The Kappa statistic was used to measure the interrater reliability of multiple annotation results. Using the above corpus, we developed a machine-learning method to automatically classify these questions into one of the following six classes: *Condition Management*, *Healthy Lifestyle*, *Diagnosis*, *Health Provider Choice*, *Treatment*, and *Epidemiology*.

**Results:**

The consumer health question schema was developed with a four-hierarchical-level of specificity, comprising 48 quaternary categories and 35 annotation rules. The 2000 sample questions were coded with 2000 major codes and 607 minor codes. Using natural language processing techniques, we expressed the Chinese questions as a set of lexical, grammatical, and semantic features. Furthermore, the effective features were selected to improve the question classification performance. From the 6-category classification results, we achieved an average precision of 91.41%, recall of 89.62%, and *F*_1_ score of 90.24%.

**Conclusions:**

In this study, we developed an automatic method to classify questions related to Chinese health care posted by the general public. It enables Artificial Intelligence (AI) agents to understand Internet users’ information needs on health care.

## Introduction

The Internet is increasingly becoming a main resource for consumers to acquire health information. Until December 2015, there were 152 million Internet health users in China, indicating that 22.1% of Chinese Internet users have looked online for health information and services [1]. Many studies have proved that health-related information online could impact consumers’ health-related attitudes and behaviors [2-4]. However, consumers have difficulty in expressing their information needs accurately using medical query terms, thus failing to retrieve relevant health information [5,6]. Automatic question answering (QA) systems are available for such users and they respond with concise and correct answers using natural language processing techniques. Thus the QA systems have become one of the most important research focuses in the field of biomedicine [7].

In general, a QA system consists of 3 modules: question analysis, information retrieval, and answer extraction. In the first module, question classification plays an important role in identifying the information needs of consumers, reducing the space of candidate answers, and further improving the accuracy of returned answers [8]. Classification schema is the basis of question classification. However, due to the difference in the information needs of health providers and consumers [9-11], the existing question classification schemas for professional health-related questions (the International Classification of Primary Care [12,13], the Taxonomies of Generic Clinical Questions (TGCQ) [14], etc) are not suitable for consumer health questions. Although some research focuses on the classification schema of consumer health questions [15,16], it has not been defined in a systematic manner yet. Therefore, it is a prerequisite to design a concise and valid classification schema.

Several studies have been conducted for automatic quesiton classification in the field of health and medicine in order to identify the general topics of clinical questions [17], distinguish between answerable and unanswerable intensive care unit (ICU) questions [18], separate consumer health questions from professional medical questions [19], and classify the types of consumer health questions [20]. Research has demonstrated that support vector machines (SVMs) performed the best among the most commonly explored algorithms, including naive bayesian, decision tree, maximum entropy, logistic regression, and conditional random fields. However, different patterns of thinking and habits of Chinese expression offen cause a mass of difference in the flexibility of word order and parse for Chinese health questions [21]. Several studies on Chinese NLP focused on clinical named entity recognition [22], diseases, or drag-related clinical information extraction [23,24] and speculation detection [25] from the free-text of pathology and operation notes. The main challenges in these tasks were word segmentation and feature representation and selection. To our knowledge, few studies have investigated consumer health question classification in Chinese.

As one of the most common chronic diseases, hypertension has become the main risk factor of cardiovascular diseases. It was estimated that China had 270 million patients with hypertension in 2012, and the incidence rate was approximately 3% per year [26]. Thus, hypertension-related questions are frequently asked with large variability on the Internet. For this reason, this study aimed at building a general topic classification schema and an automatic classification method for consumer health questions in Chinese, for the purpose of facilitating users’ hypertension-related information needs analysis and answer extraction.

## Methods

### Data Collection

We collected questions posted by health consumers from 1st January to 10th August, 2014, with the tags “hypertension (高血压)” or “blood pressure (血压)” under the Q&A (有问必答) section on a Chinese health website with more than 35 million registered users [27] and imported the data into a MySQL (MySQL Community Edition, Oracle) database. The resulting database included 98,032 messages, from which 2000 messages were randomly selected as the sample for analysis.

In this study, “question” is defined as a request that a health consumer has posted on the website on a certain subject to elicit answers from physicians, which was identified based on meaning, not form. We focused on questions related to hypertension (*高血压*), which was sometimes expressed as “high blood pressure (*高血压*),” or simply as “high pressure (*高压*).” Therefore, we manually discarded messages that did not match the definition and that were irrelevant to hypertension but which contained similar words such as “high pressure oxygen (*高压氧*),” “hyperbaric cabin (*高压舱*),” “high voltage (*高压电*),” “pressure cooker (*高压锅*),” and so on. A new message was randomly selected from the database when an irrelevant message was discarded from the sample, so as to keep the sample size at 2000.

The website provides a template for users to generate questions, which includes three fields: (1) describe your health status (*病情描述*), (2) treatments or tests in the past (*曾经的治疗或检查情况*), and (3) what kinds of help do you want (*想得到怎样的帮助*). This template might lead to confusion in customers regarding how to post their questions. To deal with this case, we developed a rule: if the phrase “what kinds of help do you want (*想得到怎样的帮助*)” was found in the message, then we would take the sentence after the phrase as the “question.” Otherwise, we would take the whole message as the “question.” By doing this, we collected 2000 questions with an average length of 48 words.

### Classification Schema and Corpus Construction

**Figure 1:**
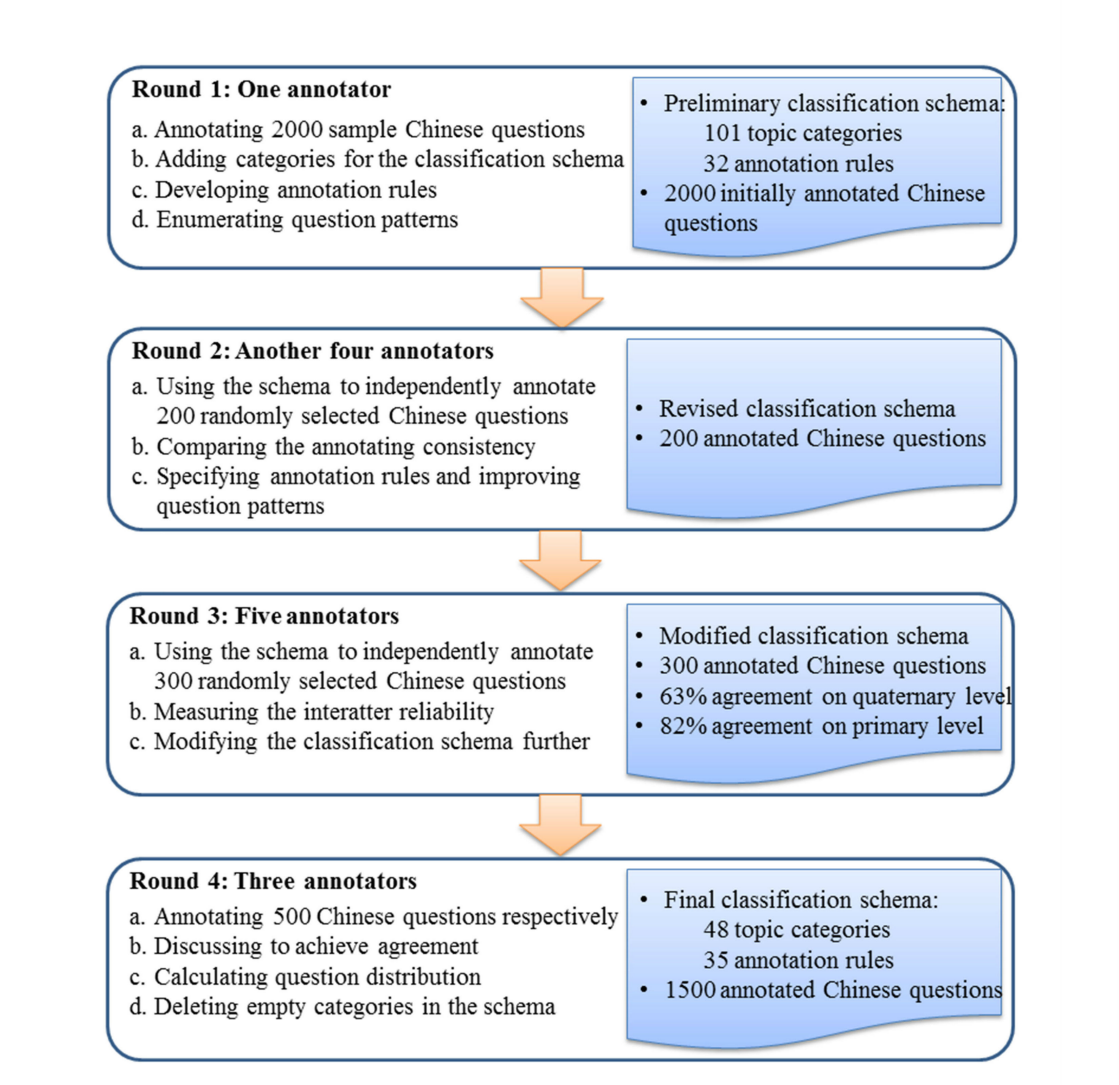
A four-round annotating process to construct and modify the classification schema and annotated corpus.

A topic-based classification schema was developed based on TGCQ [14] and the Layered Model of Context for Consumer Health Information Searching (LMCC) [15], and some categories were divided into more specific sub-categories to code the specific information needs. We produced the annotations in 4 rounds (Figure 1). In round 1, one annotator (specialized in medical informatics) annotated all the 2000 sample questions, following the classification schema. Some categories were added to accommodate questions that did not fall into any existing specialty during the process. For purposes of consistency improvement among coders, and the usability of the classification, a list of annotation rules was developed and some question patterns were enumerated for even the smallest category. As a result, the preliminary classification schema for consumer health questions included 101 topic categories and 32 annotation rules.

In round 2, four other annotators (two specialized in medicine and two specialized in informatics) independently annotated 200 questions randomly selected from the sample, using the classification schema. The authors compared the consistency of the five coding results (including the one in the first round) and categorized the 200 questions into three groups: (1) all annotators agreed (n=73), (2) only one disagreed (n=63), and (3) more than one disagreed (n=64). Then we focused on the last group. We addressed ambiguous elements by further specifying annotation rules and improving the descriptions of the question patterns.

In round 3, the revised classification was distributed to the five annotators who independently annotated another 300 questions randomly selected from the remaining sample of 1800 messages. This step was done to measure the interrater reliability of the classification schema as well as to further modify it.

In the last round, each of three annotators independently annotated 500 from the remaining 1500 messages. So each of the 2000 sample questions were annotated by at least two annotators. The authors compared the coding results and the disparities were discussed to achieve an agreement. The codes agreed upon during this step were regarded as the final schema. The number of questions in each category was calculated, and categories in which no questions were filled were deleted (such as physical characteristics of drugs, pharmacodynamics, and mechanism of drug action).

### Automatic Classification of the First-Level Topics

The 2000 questions annotated by the above steps were used to train and test the classifiers for the primary level topics, including *Diagnosis*, *Treatment*, *Condition Management*, *Healthy Lifestyle*, and *Health Provider Choice*.

#### Feature Selection

We explored various features for machine-learning, including lexical, grammatical, semantic, and statistical information (Multimedia Appendix 1).

##### Lexical Features Such as Bag-of-Words and Part-of-Speech

The word segmentation was obtained from Rwordseg [28], a Chinese word segmentation tool under R environment based on the Chinese lexical analysis system, ICTCLAS, which reached a precision of 97.58% on the *973* experts testing [29]. The Chinese part-of-speech tag was obtained by using the Stanford Parser (version 3.3.1) [30].

##### Grammatical Features Such as Interrogative Words, Noun Head Chunks, Verb Head Chunks, Noun Rear Chunks, Verb Rear Chunks, Interrogative Words + Noun or Verb Head Chunks, and Noun or Verb Rear Chunks + Interrogative Words

We manually developed a dictionary of 42 Chinese interrogative words based on baike.baidu [31] and general types of Chinese consumer questions summarized by our former research [32]. In this study, the noun or verb head chunk is the first noun or verb after the first interrogative word in a question, and the noun or verb rear chunk is the last noun or verb before the first interrogative word. They are likely to be the dependent words of the interrogative word that help to express the semantic information in the question [33].

##### CMeSH Concepts and Semantic Types

The controlled vocabulary of Chinese Medical Subject Headings (CMeSH) [34] was applied to recognize the medical concepts and their semantic types (*Disease*, *Drug*, and *Symptom*) in the Chinese consumer health questions.

##### Keywords

These were a combination of lexical and statistical features. We used three ways to extract the keywords from a question: (1) the first *k* words of maximum term frequency (TF), (2) the first *k* words of maximum inverse-document frequency (IDF), and (3) the first *k* words of maximum TF-IDF. We adopted the heuristic equation (1) developed by Cao et al [17] to calculate *k*, which was based on the observation that the number of keywords increases when the question length increases.

##### Statistical Features

**Figure 2:**
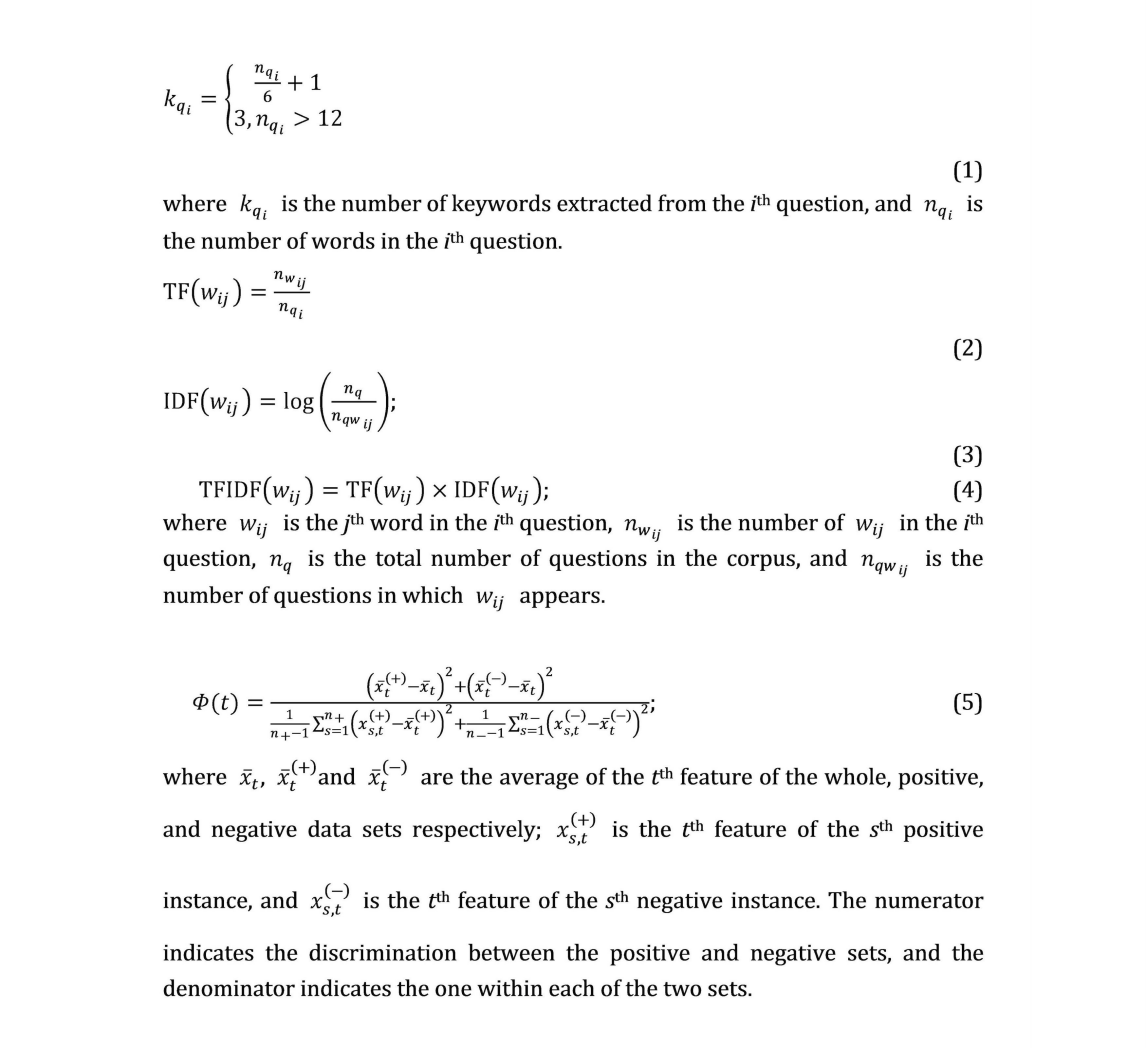
Mathematical equations.

These include question length, maximum, minimum, and average word length, maximum, minimum and average TF, maximum, minimum, and average IDF, and maximum, minimum and average TF-IDF. The corpus used to calculate the IDF of each word contained nearly 100 thousand hypertension-related messages that we had collected in our former research [32]. The TF, IDF, and TF-IDF were computed by equations (2), (3), and (4), shown in Figure 2.

As the feature space dimension was very large, and some of them could have degraded the performance of the classifiers, we adopted Φ-score to select the most discriminative features, which measures the discriminations in two sets of real numbers [35]. Given the training vectors x_s,_, s=1,..., m, if the number of positive and negative instances are n+ and n-, respectively, the Φ-score of the t^th^ feature is defined in equation (5) in Figure 2. The larger the Φ(t) is, the more likely this feature is more discriminative. Therefore, we used Φ(t) as the feature selection criterion, and the implementation steps were as follows:

Calculate Φ(t) of every feature

Calculate the avg Φ of each type of feature and, further, set it as the threshold of the corresponding feature type. The avg Φ was chosen as the feature selection threshold because the distribution of Φ differs greatly between different types of features, while this method can help to keep all the useful features in different types [36].

For each type of machine-learning feature, select features with Φ ≥ avg Φ of this type.

#### Classifiers

Since a question can be assigned to multiple topics, the task in this paper was a multi-label classification problem, which was usually transformed into one or more single-label classification or regression problems [37]. We therefore transformed the task into six binary classification problems (one-versus-rest for each) so as to suit the SVMs [38], which were commonly used and claimed to be the best in related works [17-20]. We used machine-learning algorithms within the R project for statistical computing (version 3.3.1) for automatic question classification, including naive bayesian, SVMs, decision tree, maximum entropy, logistic regression, and conditional random fields. The results showed that SVMs performed the best among all the algorithms in 10-fold cross-validation.

#### Training and Testing

Due to the skewed distribution of consumer questions to different topics, an under-sampling method for the majority classes was applied to ensure that each classifier was trained and tested on the same number of “positive” and “negative” questions. We reported the classification performance using 10-fold cross-validation. The sample data for each binary classifier was equally divided into 10-folds: one of them was used as testing data, and the ramaining 9 folds as training data. The cross-validation process was repeated 10 times (equal to the folds) and the average value and standard deviation were reported. All cases in the sample data were used for both training and validation. Thus, each case was used for validation exactly once, which was the distinct advantage in this method [39].

### Evaluation Metrics

The interrater reliability of the classification schema was evaluated by the kappa statistic, which could correct agreement that occurred by chance. Kappa=(P_o_-P_e_)/(1-P_e_), where P_o_ is the observed agreement and P_e_ is the agreement expected by chance [40]. When the number of categories was large, as in this study, P_e_ would be close to zero, and the kappa value would be close to P_o_ [14]. Thus, we directly used P_o_ as the kappa value. The bigger the kappa value, the better the agreement. We assume that when the user asked more than one question, it was acceptable to answer any one of them. Therefore, a liberal reliability criterion was used: a match was recorded if either the main or minor topics assigned by one annotator matched the other’s assignments.

The performance of automatic classification methods was evaluated by precision (p), recall (r) and *F*_1_ score, all of which are commonly used as evaluation metrics for text categorization, and we report the average of each metric. Precision is the number of correctly classified cases divided by the total number of cases classified for the category; recall is the number of correctly classified cases divided by the total number of cases of that class; and *F*_1_ score is the harmonic mean of precision and recall, calculated as *F*_1_ =(2×p×r) / (p + r).

## Results

### Classification Schema of Consumer Health Questions

The final classification schema was a four-hierarchical-level of specificity, consisting of 48 quaternary categories (see Multimedia Appendix 1) and 35 annotation rules. The first level included seven areas, namely diagnosis, treatment, condition management, epidemiology, healthy lifestyle, health provider choice, and other. A branching structure of secondary, tertiary, and quaternary levels describes more specific topics of the questions than its upper level. One or more closely related question patterns were listed for each quaternary category. Table 1 shows examples of consumer health questions in Chinese with their pattern and annotated tags on the topics of diagnosis and treatment, respectively.

Table1. An example of consumer health questions in Chinese with their pattern and annotated tags.

**Table:** 

General Topics	Items	Contents
Diagnosis	Question	昨天不知道怎么事，突然感到心慌慌的，四肢发凉，全身冒冷汗，之后老婆扶我到小区医院那里去看，量了一下血压，血压比以往要高，之后医生叫我放松，休息了20分钟左右，又感觉没有什么事了。。 请问突然感觉到心慌，四肢发凉，血压升高，这是啥病啊? (Yesterday, my heart suddenly palpitated, my limbs became cold, and my whole body began to sweat. Then my wife accompanied me to the community hospital and checked my blood pressure; it was higher than before. The doctor told me to relax, and I feel much better after resting for about 20 minutes… suddenly felt flustered, limbs became cold, and blood pressure rose. What disease is it?)
	Pattern	临床发现X1、X2、X3、……，这是啥病？(Clinical finding X1, X2, X3,… What disease is it?)
	Tag	1.1.4.1 “诊断(Diagnosis)→病因/临床发现的解释(Interpretation of clinical finding)→不具体的发现或多种发现(Uncertain/multiple findings)”
Treatment	Question	65岁老人血压高经常不稳定，吃哪种降压药最好？(A 65-year-old man with unsteady high blood pressure… What’s the best blood pressure drug to take?)
	Pattern	病情y，吃/用/服用哪种药最好？(Condition y: What’s the best drug to take or use?)
	Tag	2.1.2.1 “治疗(Treatment)→药物治疗(Drug therapy)→效力/适应症/药物选择(efficacy/indications/drug choosing)→治疗(Treatment)”

### General Topics of Questions Asked by Health Consumers

This study found that although health consumers would ask numerous health questions about themselves or their families, the general topics of the questions were limited to a small number and each category of the topics had its particular question patterns. The 2000 Chinese consumer health questions were annotated with 2000 major codes and 607 minor codes. The distribution of the sample questions on the primary level category is shown in Table 2. 26.35% of the questions were annotated with more than one topic, which demonstrated that health consumers tend to ask more than one question at a time and the question messages usually belong to multiple topic categories [11] (Multimedia Appendix 1). These findings indicated that the various consumer health questions could be represented by limited topics and keywords, and the task to classify those topics was a multi-label problem [37].

**Table 2:** Distribution of the 2000 consumer health questions in Chinese on the primary level of topics.

No.	General Topics	Positive	Negative	Total
1	Diagnosis	600	1400	2000
2	Treatment	1167	833	2000
3	Condition management	136	1864	2000
4	Epidemiology	233	1767	2000
5	Healthy lifestyle	278	1722	2000
6	Health provider choice	45	1955	2000
7	Other	5		
	Total	2000	2000	2000

### Interrater Reliability of the Classification Schema

The kappa statistic for the five annotators was 0.63 in the quaternary level of the classification, indicating “substantial” reliability, better than in several similar studies, such as assigning topics to general clinical questions (kappa=0.53) [14]. When only the primary and secondary levels were considered, the kappa value increased to 0.75. When only the seven broad areas in the primary level were considered, the kappa value was 0.82, slightly better than automatically classifying question types for consumer health questions in English conducted by Roberts et al [20].

### Feature Selection for Automatic Question Classification

The Φ-score of each feature was calculated for each binary classifier. We found that their distribution between different types of features differed greatly. The performance of classifiers using features with Φ ≥ avg Φ was not worse than that of those classifiers using all the features in the corresponding types, and some of them were even higher than the latter. Taking the topic of *Lifestyle* as an example, the average and standard deviation of Φ in each feature type are shown in the third and fourth columns in Table 3. The avg Φ of bag-of-words was 0.0016 with a standard deviation of 0.0067, while the values of keywords with maximum TF were 0.0008 and 0.0009, respectively. The average *F*_1_ score of the classifier was 74.08% when using all the 6154 features in part-of-speech, while the performance increased to 78.84% when just taking the 1490 features with Φ ≥ avg Φ (Figure 2). Similar cases can be seen in feature types of noun rear chunks, interrogative + noun or verb head chunks, verb rear chunks + interrogative, keywords with maximum TF, TF-IDF, and so on. The observations indicated that some of the features in each type either do nothing to the classifiers or have some side effects on them. Rejecting these features could not only save the computing resources so as to increase the efficiency, but also improve the performance of the classifiers.

**Table 3:** Number and Φ distribution of each type of feature for the Chinese consumer health question classification on the topic of *Lifestyle.*

Levels	Features Types^a^	Avg Φ	σ (Φ)	n_AF_	n_(Φ ≥ avg Φ)_
Lexical	Bag-of-words	0.0016	0.0067	4967	1301
	Part-of-speech	0.0014	0.0060	6154	1490
Grammatical	Interrogative words	0.0039	0.0204	97	13
	Noun head chunks	0.0011	0.0010	48	14
	Verb head chunks	0.0008	0.0007	19	6
	Noun rear chunks	0.0011	0.0019	73	14
	Verb rear chunks	0.0010	0.0013	22	3
	Interrogative + noun head chunks	0.0011	0.0013	328	86
	Interrogative + verb head chunks	0.0011	0.0010	312	85
	Noun rear chunks + interrogative	0.0010	0.0013	315	67
	Verb rear chunks + interrogative	0.0012	0.0024	318	74
Semantic	CMeSH concepts	0.0016	0.0033	43	9
	CMeSH semantic types	0.0124	0.0101	3	1
Lexical & Statistical	Keywords (TF)	0.0008	0.0009	1510	282
	Keywords (IDF)	0.0007	0.0008	1137	192
	Keywords (TF-IDF)	0.0008	0.0008	1208	190
Statistical	Statistical features	0.0073	0.0060	13	5
	Total with duplicates replaced			15349	3656

^a^For each type of feature, σ (Φ) is the standard deviation of Φ, n_AF_ is the total number of features, n (Φ ≥ avg Φ) is the number of features with Φ ≥ avg Φ.

Therefore, the features with Φ ≥ avg Φ in every feature type were selected as input features for machine-learning, in order to keep all the useful features in different types and to improve the performance of the classifiers. Thus, each classifier received a different feature set, and the number of features within them are showed in the third column in Table 4. For example, words such as “*drinking* (饮 酒),” “*eat a meal* (吃 饭),” “*breakfast* (早餐),” “*stay up late* (熬夜),” “*weight* (体重),” “*daily life* (平时),” “*nurse one’s health* (调理),” and so on were the effective bag-of-words features for the classifier for *Healthy Lifestyle* but not effective for the classifier for *Diagnosis*. On the contrary, words such as “*diagnose* (诊断),” “*judge* (判断),” “*indicate* (提示),” “*physical examination* (查体),” “*cardiac murmur* (杂音),” “*head rush* (脑充血),” “*dazed* (昏沉沉),” and so on were the effective bag-of-words features for the classifier for *Diagnosis* but not effective for the classifier for *Healthy Lifestyle*.

**Table 4:** Feature reduction and the performance of each classifier.

General topics	N (all features)	N (selected features)	Feature reduction proportion	Avg *F*_1_	σ (*F*_1_)
Diagnosis	15349	5311	0.6540	0.9855	0.0164
Treatment	15349	4216	0.7253	0.7602	0.0482
Condition management	15349	3150	0.7948	0.9963	0.0117
Epidemiology	15349	4194	0.7268	0.7177	0.0798
Healthy lifestyle	15349	3656	0.7618	0.9913	0.0166
Health provider choice	15349	2282	0.8513	0.9635	0.0594

**Figure 3:**
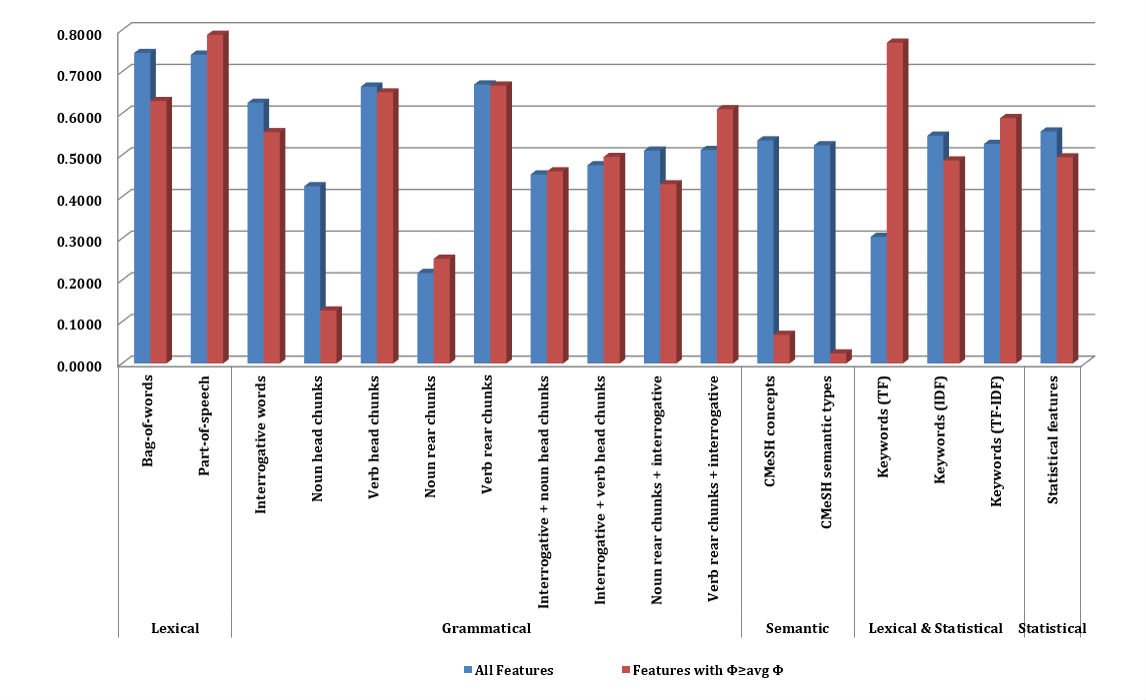
Performance of each feature type for Chinese consumer health question classification on the topic of Lifestyle.

**Figure 4:**
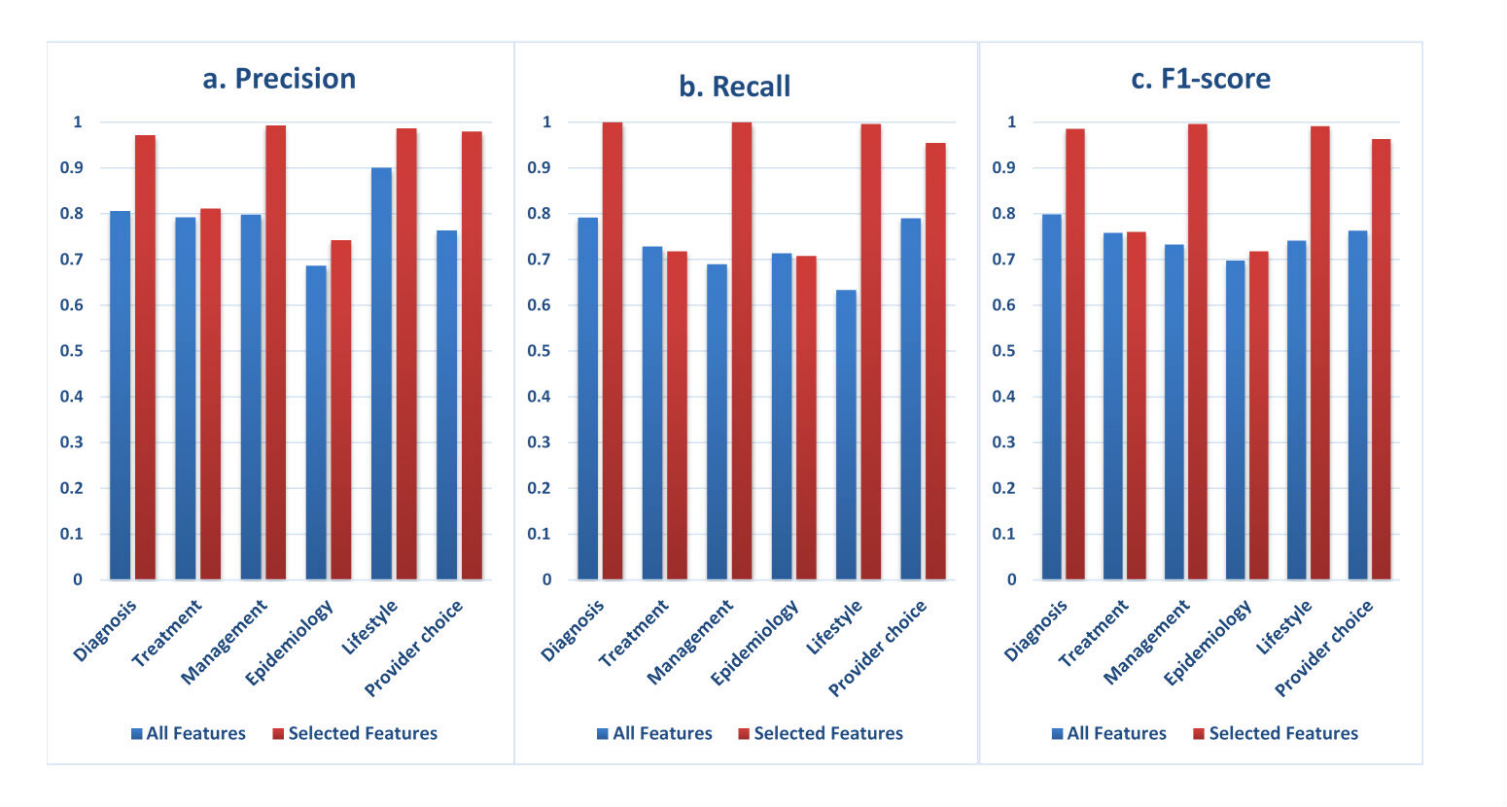
Performance improvement of each classifier by selecting features above the threshold.

### Performance of the Automatic Classification Methods

The results were obtained from SVMs in the kernlab package because it performed the best among all the classification algorithms available in the R project. The research findings showed that the feature spaces were reduced from 65.40% to 85.13% by dropping features under the threshold (Table 4). Furthermore, the performance of each classifier also improved significantly (Figure 3). For example, the average *F*_1_ score of the classifier for *Diagnosis* dramatically increased from 79.84% to 98.55%, while the feature space reduced from 15349 to 5311 (reduced 65.40%). The feature space of the classifier for Treatment reduced greatly (Down from 15349 to 4216, reduced 72.53%), although the improvement of performance was not so obvious (F1 score increased from 75.82% to 76.02%). The results of 10-fold cross validation on each binary classifier affirmed the hypothesis we proposed in the Feature Selection sections.

The performance on the classification of most topics of consumer health questions in Chinese was high. The evaluation metrics (average precision, recall, and *F*_1_ score) of *Diagnosis, Condition Management, Healthy Lifestyle, and Health Provider Choice* were above 95%, while, the metrics of *Treatment* and *Epidemiology* were 76.02% and 71.77%, which were relatively weaker than the others. The standard deviation indicated that the performance of each binary classifier was relatively robust. As of 6-category classification results, we achieved an average precision of 91.41%, recall of 89.62%, and *F*_1_ score of 90.24%.

## Discussion

### Principal Findings

A classification schema of consumer health questions was built in this study and 2000 hypertension-related consumer health questions in Chinese were manually annotated based on this schema. The research findings demonstrated that health consumers were mainly concerned about what was wrong with their health (or the health of someone they cared about), why it was wrong, how to treat it (including choosing which provider to treat), whether the drugs they used had adverse effects or would do harm in some conditions (eg, pregnancy, breast feeding), whether they could recover from the illness, and what they could do to improve their health in everyday life (mainly diet suggestions).

We explored a machine-learning method to automatically classify these Chinese consumer health questions into one of the six primary level topics, with a novel scoring metric to select the most effective features from the abundant feature types we had explored. The results proved that selecting the features with Φ ≥ avg Φ in each feature type as input features for machine- learning not only increased the efficiency, but also improved the performance of the classifiers successfully. From the 6-category classification results, we achieved an average precision of 91.41%, recall of 89.62%, and *F*_1_ score of 90.24%.

### Comparison With Prior Work

#### Similarities and Differences in Questions Asked by Health Consumers and Providers

Compared with the 1396 clinical questions annotated by Ely et al [14,41], we found that while health consumers and providers both asked questions about diagnosis, treatment, condition management, and epidemiology, the questions posted by consumers were much more ambiguous. For instance, the frequency of questions with multiple findings was twice that of health providers’ inquiries under the category of interpretation of clinical findings. It might be because consumers could not identify the most important findings, so they tended to list all the findings they knew. Although the frequency of treatment questions was almost equal in the two groups, health providers’ questions were more specific to drug therapy (37.2% vs 22.1%), and they sometimes asked these questions on very specialized topics, such as composition, pharmacodynamics, action mechanism, and serum levels of drugs [14,41]. Such questions were rarely asked by health consumers. Moreover, health consumers would ask how to keep healthy or help in recovery in daily life, because many of them have recognized that lifestyle factors, such as diet, exercise, weight loss, and mood control, would impact their health status as well [42]. However, physicians seldom asked these questions during a patient encounter, possibly because they mainly focused on medical service rather than lifestyle advice [10]. Similarly, health consumers never asked questions about coordination with other providers, doctor-patient communication, doctor and patient education, administrative rules, ethics, and legal issues, because these tasks were usually regarded as health providers' responsibility. These findings affirmed again that, health consumers’ information needs differed significantly from those of providers. Therefore, the existing classification schemas and automatic classification methods for clinical questions cannot be applied to consumer health questions directly [11].

#### Features Explored for Automatic Classifiers

Compared with other related studies on automatic question classification in the domain of health and medicine, we explored an abundant number of feature types for automatic classifiers. For example, Cao et al [17] explored the features of bag-of-words, n-grams, part-of-speech, UMLS concepts, and semantic types, as well as IDF to identify general topics of clinical questions. Patrick et al [18] used bag-of-words, Bigram, interrogative words, SNOMED category, verb and its subject, and verb and its object as feature sets to distinguish answerable and unanswerable ICU questions. Liu et al [19] picked bag-of-words, word length, question length, IDF, interrogative words, personal pronouns, indefinite pronouns, and auxiliary verbs as learning features to separate consumer questions from professional questions in the health domain. Roberts et al [20] explored the features of bag-of-words, part-of-speech, UMLS concepts, named entity, word length, IDF, and noun and verb head chunks to classify question types for consumer health questions in English, while Conway et al [43] used bag-of-words, n-grams, semantic UMLS types, and named entity as features to classify disease outbreak reports. In other words, bag-of-words, part-of-speech, and semantic types were the most commonly used features for question classification. Our work adopted all the effective features in the prior works with the UMLS concepts and semantic types replaced by CMeSH concepts and semantic types. We also explored three ways to extract the keywords from a question using the machine-learning features, that is, we took the first *k* words with maximum TF, IDF, or TF-IDF as keywords. In addition, we added noun or verb rear chunks + interrogative words according to the specialties of word order in the Chinese language. The results showed that it worked better than the interrogative words + noun or verb head chunks, which was commonly used for question classification in English.

#### Feature Selection for Automatic Question Classification

The feature selection methods in our work were quite different from other relative works, and it has proved that our methods were much more effective and easy. Cao et al [17], Liu et al [19], and Roberts et al [20]employed a method of combining different types of features without considering the threshold, in which they explored different combinations of different feature types and selected the best combination with the maximum *F*_1_ score of the classifier. Thus, a feature type would be either picked up or rejected in their approach, which may have caused the loss of some effective features. Another disadvantage of this method was the difficulty in exploring all the possible combinations of different feature types. On the contrary, we adopted a much more efficient method to combine all the effective features from each feature type with Φ ≥ avg Φ, which was also proved to be very effective (as described in the results section).

#### Performance of the Classifiers

The performance of the classifiers trained by our study was quite satisfying. The average *F*_1_ scores for the four classifiers for *Condition management, Health lifestyle, Diagnosis, and Health provider choice* were 99.63%, 99.13%, 98.55%, and 96.35%, respectively. The results were more significant than those of other similar studies. For example, the classification of 13 general topics of clinical questions conducted by Cao et al [17], which reached the highest *F*_1_ score (89.3%) on the classifier for *Pharmacology*, while the majority of the scores were between 70% and 80%, and the classification of 13 question types of consumer questions carried out by Roberts et al [20], which achieved the highest *F*_1_ score (90.6%) on the classifier for *Management*, with 5 between 80% and 90%, and 5 below 70%. It is worth noting that the methods proposed in this paper and those in the related works were experimented on different datasets in different languages. Further, there were two main reasons for the differences between this study and others, although the same algorithm of SVMs was used. On the one hand, the feature types and the feature selection methods applied in this study were more efficient and effective (as discussed above); on the other hand, the classification schema used in this study was more distinguishable. For example, questions about *Etiology* or *Cause* and *Diagnosis* were very similar and always asked together. Thus, they were annotated as one topic (*Diagnosis)* in our study. However, Cao et al [17] and Roberts et al [20] annotated them as different types.

### Limitations

One of the limitations of this work is that the sample questions we used to build the classification schema and to train the automatic classifiers were from only one Chinese health website and defined to be hypertension or blood pressure related. Therefore, the applicability of the classification schema and the validity of the automatic classifiers for the vast majority of questions from other websites and other diseases remain to be tested. Another limitation of this work is that some types of features, such as keywords and bag-of-words, might be correlated. However, our feature selection algorithm did not take the impact of correlation into consideration. We only reached moderate performances on the automatic classifiers for the general topics of Treatment and Epidemiology, whereas the reasons for this remain to be explored in the future.

### Conclusions

One of the specialties of this research was that Chinese consumer health questions were chosen as the research object. We built a classification schema of consumer health questions which consisted of 48 quaternary categories and 35 annotation rules, and we annotated 2000 questions in Chinese that were randomly selected from nearly 100 thousand messages about hypertension. Then, by using these annotated questions as the corpus, we explored a machine-learning method to automatically classify Chinese consumer health questions into six general topics to facilitate users’ information needs analysis and answer extraction. We explored an abundant number of feature types and adopted a novel method to select all the effective features with Φ ≥ avg Φ. The results proved that our classification approach was relatively more efficient and effective as compared with similar studies.

## Appendix

Multimedia Appendix 1 Supplementary tables.

## Abbreviations

TGCQ: Taxonomies of Generic Clinical Questions
LMCC: Layered Model of Context for Consumer Health Information Searching
