# Supplementary material for: Classifying Chinese Questions Related to Health Care Posted by Consumers Via the Internet
Source: J Med Internet Res. 2017 Jun 20;19(6):e220. doi: 10.2196/jmir.7156 (PMC5497072; doi:10.2196/jmir.7156)
Supplement: Multimedia Appendix 1 [file jmir_v19i6e220_app1.pdf]

Appendix

Table a. An example of all the features considering for a consumer health question in Chinese

	Content		
**First Level Topics**	Diagnosis		
**Sample question**	76 [illegible]		
**Lexical Features**			
(Bag-of-words)	76 [illegible]		
(Part-of-speech)	76 [illegible]#NR [illegible]#NN [illegible]#DT [illegible]#NN [illegible]#VV [illegible]#LC [illegible]#VV [illegible]#AD [illegible]#VV [illegible]#NN [illegible]#VV [illegible]#VV [illegible]#VV [illegible]#AD [illegible]#VV [illegible]#AD [illegible]#VV [illegible]#NN [illegible]#AD [illegible]#VC [illegible]#JJ [illegible]#M [illegible]#NN [illegible]#PN [illegible]#NN [illegible]#VV [illegible]#VE [illegible]#NN [illegible]#NN [illegible]#NN [illegible]#PN [illegible]#AD [illegible]#VC [illegible]#NT [illegible]#NT [illegible]#CC [illegible]#NT [illegible]#VC [illegible]#VA [illegible]#DEC [illegible]#JJ [illegible]#DEG [illegible]#NN [illegible]#VC [illegible]#JJ [illegible]#M [illegible]#NN [illegible]#SP		
**Grammatical Features**			
(Interrogative words)	[illegible]#JJ [illegible]#JJ [illegible]#SP		
(Noun head chunks)	[illegible]	(Interrogative + noun head chunks)	[illegible]
(Verb head chunks)	[illegible]	(Interrogative + verb head chunks)	[illegible]
(Noun rear chunks)	[illegible]	(Noun rear chunks + interrogative)	[illegible]
(Verb rear chunks)	[illegible]	(Verb rear chunks + interrogative)	[illegible]
**Semantic Features**			
(CMeSH concepts)	[illegible](Hypertension, MeSH ID: D006973)<br>[illegible](Diabetes Mellitus, MeSH ID: D003920)<br>[illegible](Coronary Disease, MeSH ID: D003327)		
(Semantic types)	dis dis dis		
**Lexical & Statistical Features**			
(Keywords (TF))	[illegible]		
(Keywords (IDF))	[illegible] 76[illegible] [illegible]		
(Keywords (TF-IDF))	[illegible] [illegible] 76[illegible]		
**Statistical Features**			
(Question length)	77	(max IDF)	6.9980
(max word length)	3	(min IDF)	0.8619
(min word length)	1	(average IDF)	3.2017
(average word length)	1.5385	(max TF-IDF)	0.1460
(max TF)	0.0833	(min TF-IDF)	0.0212
(min TF)	0.0208	(average TF-IDF)	0.0778
(average TF)	0.0272		

Table b. Classification schema of consumer health questions

Code	Primary	Secondary	Tertiary	Quaternary
1.1.1.1	diagnosis	etiology/interpretatio	symptom	[illegible]

Code	Primary	Secondary	Tertiary	Quaternary
1.1.2.1		n of clinical finding	sign	□
1.1.3.1			test finding	□
1.1.4.1			uncertain/multiple findings	
1.2.1.1		criteria		
1.3.1.1		test	indications/ efficacy	□
1.3.2.1			accuracy	□
1.3.3.1			timing	□
1.3.4.1			method	
1.4.1.1		orientation	condition	□
1.5.1.1		cost	□	□
2.1.1.1	treatment	drug therapy	how to use	general
2.1.1.2				dosage
2.1.1.3				timing
2.1.2.1			efficacy/ indications	treatment
2.1.2.2				prevention
2.1.3.1			adverse effects	caused by drug
2.1.3.2				control
2.1.3.3				safety/contraindication
2.1.4.1			interactions	□
2.1.5.1			name	□
2.1.6.1			cost	□
2.1.7.1			availability	□
2.1.8.1			brand	□
2.2.1.1		not limited to but may include drug therapy	efficacy/ indications	treatment
2.2.1.2				prevention
2.2.2.1			timing	□
2.2.3.1			how to do it	□
2.2.4.1			safety/contra/sequela	□
2.2.5.1			cost	□
3.1.1.1	management	condition	□	
4.1.1.1	epidemiology	prevalence		
4.2.1.1		etiology	causation/ association	risk factors
4.2.1.2				genetics
4.3.1.1		prognosis	□	□
5.1.1.1	healthy lifestyle	diet	how to eat	□
5.1.2.1			food choosing	efficacy
5.1.2.2				contraindication
5.1.3.1			interactions	□
5.1.4.1			general	□
5.2.1.1		exercise	□	□
5.3.1.1		weight-losing	□	□

Code	Primary	Secondary	Tertiary	Quaternary
5.4.1.1		mood control	[illegible]	[illegible]
5.5.1.1		general	[illegible]	[illegible]
6.1.1.1	health provider choosing	hospital	[illegible]	[illegible]
6.2.1.1		department	[illegible]	[illegible]
6.3.1.1		doctor	[illegible]	[illegible]
7.1.1.1	other	[illegible]	[illegible]	[illegible]

Textbox 1. An example of asking more than one questions by health consumer at a time

Question Message	Main Code	Minor Codes
[illegible] 48 [illegible] 1 [illegible]	2.2.1.1	5.5.1.1<br>5.7.1.1<br>4.2.1.2
